# Gene expression and epigenetic responses of the marine Cladoceran, *Evadne nordmanni*, and the copepod, *Acartia clausi*, to elevated CO_2_

**DOI:** 10.1002/ece3.8309

**Published:** 2021-11-23

**Authors:** Neelakanteswar Aluru, David M. Fields, Steven Shema, Anne Berit Skiftesvik, Howard I. Browman

**Affiliations:** ^1^ Biology Department Woods Hole Oceanographic Institution Woods Hole Massachusetts USA; ^2^ Bigelow Laboratory for Ocean Sciences East Boothbay Maine USA; ^3^ Institute of Marine Research Austevoll Research Station, Ecosystem Acoustics Group Storebø Norway

**Keywords:** climate change, DNA methylation, ocean acidification, marine cladocerans, RNAsequencing

## Abstract

Characterizing the capacity of marine organisms to adapt to climate change related drivers (e.g., pCO_2_ and temperature), and the possible rate of this adaptation, is required to assess their resilience (or lack thereof) to these drivers. Several studies have hypothesized that epigenetic markers such as DNA methylation, histone modifications and noncoding RNAs, act as drivers of adaptation in marine organisms, especially corals. However, this hypothesis has not been tested in zooplankton, a keystone organism in marine food webs. The objective of this study is to test the hypothesis that acute ocean acidification (OA) exposure alters DNA methylation in two zooplanktonic species—copepods (*Acartia clausii*) and cladocerans (*Evadne nordmanii*). We exposed these two species to near‐future OA conditions (400 and 900 ppm pCO_2_) for 24 h and assessed transcriptional and DNA methylation patterns using RNA sequencing and Reduced Representation Bisulfite Sequencing (RRBS). OA exposure caused differential expression of genes associated with energy metabolism, cytoskeletal and extracellular matrix functions, hypoxia and one‐carbon metabolism. Similarly, OA exposure also caused altered DNA methylation patterns in both species but the effect of these changes on gene expression and physiological effects remains to be determined. The results from this study form the basis for studies investigating the potential role of epigenetic mechanisms in OA induced phenotypic plasticity and/or adaptive responses in zooplanktonic organisms.

## Graphical abstract

Characterizing the capacity of marine organisms to adapt to climate‐change‐related drivers (e.g. pCO_2_ and temperature), and the possible rate of this adaptation, is required to assess their resilience (or lack thereof) to these drivers. In an effort to understand these, we conducted a study investigating the potential epigenetic changes in response to Ocean acidification in two planktonic species of ecological importance (Copepod, *Acartia clausi* and Cladoceran, *Evadne nordmanni*). Our results suggest that these organisms can employ epigenetic mechanisms in coping with acute changes in pCO_2_ levels. Future research will characterize long‐term adaptive responses to climatic factors such as pCO_2_ and temperature.

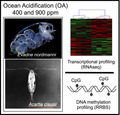

## 1 INTRODUCTION

The release of anthropogenic carbon emissions into the earth's atmosphere has resulted in changes in oceanic temperature and pH that have an impact on marine organisms and ecosystems (IPCC, 2021). IPCC scenarios project that *p*CO_2_ will continue to climb resulting in an average sea surface temperature increase of 0.6°C–2.0°C and a pH drop of 0.06–0.32 units by the end of the century (IPCC, 2013). The rapid changes in these concurrent stressors is causing concern that some marine organisms may be unable to adapt and that this will reduce the resilience of marine ecosystems in which complex food webs maintain stability (Gledhill et al., 2015).

The impacts of ocean acidification (OA) on marine organisms, are complex and species‐specific (Fabry et al., 2008; Kroeker et al., 2013; Kurihara, 2008; Wittmann & Pörtner, 2013). As genomic analysis techniques increase in efficiency, transcriptomic analyses have become important metrics for quantifying the expression of stress‐related genes (Evans & Hofmann, 2012), and enable the examination of a broad range of genetic responses to environmental change (Harms et al., 2014; Todgham & Hofmann, 2009). These techniques increase our understanding of the scope of the organismal response and have the potential to detect molecular compensation for environmental stress that may otherwise go undetected using more traditional physiological studies (Gracey, 2007). For example, in the copepod *Calanus glacialis* exposure to high pCO_2_‐low pH significantly alters genes associated with cellular stress response, oxidative stress and ion transporters, suggesting an important role for these genes in pH homeostasis (Bailey et al., 2017). Similar transcriptional responses have been reported in many other species (Strader et al., 2020). OA conditions alter DNA methylation patterns in several species of invertebrates (Downey‐Wall et al., 2020; Liew et al., 2018; Putnam et al., 2016). However, there is no experimental evidence demonstrating a direct association between molecular changes and physiological/morphological phenotypes.

The regulation of eukaryotic gene expression occurs through many pathways, including but not limited to epigenetic mechanisms such as DNA regulation and histone modifications (Gibney & Nolan, 2010). Methylation of genomic DNA provides a mechanism to modulate transcription by influencing the binding of regulatory factors to regulatory elements (Medvedeva et al., 2014). In vertebrates, DNA methylation is involved in the regulation of gene expression during cellular differentiation and development (Law & Jacobsen, 2010). Methylation of CpG dinucleotides, particularly in the promoter regions immediately upstream of the transcription start site, play a major role in the regulation of gene expression (Suzuki & Bird, 2008). More recently, it has become clear that DNA methylation in other genomic regions, such as intergenic and gene body methylation, also play important roles in gene regulation (Jeziorska et al., 2017; Zhou et al., 2020). In insects, DNA methylation is restricted to the transcribed regions and plays an important role in behavioral plasticity and social behavior (Yan et al., 2014). Research on DNA methylation is rare in crustaceans and has focused largely on the freshwater cladoceran, *Daphnia* spp. (Kvist et al., 2020; Kvist et al., 2018; Lindeman et al., 2019).

The objective of this study is to investigate the effect of acute exposure to OA on transcriptional and epigenetic (DNA methylation) patterns in two marine planktonic crustaceans, the copepod *Acartia clausi* and the Cladoceran *Evadne nordmanni*. While copepods reproduce sexually, Cladocerans have both sexual and parthenogenic reproductive pathways. Cladocerans are widely used as models to study the evolutionary basis of phenotypic plasticity because they reproduce clonally (asexually) and sexually, which offers a unique opportunity to assess the relative contributions of the epigenetic (in clonal populations) and genetic (in sexually reproducing populations) mechanisms underlying adaptation to environmental drivers and their molecular basis (Harris et al., 2012; Kvist et al., 2018; Lindeman et al., 2019; Toyota et al., 2019). By comparing the responses of copepods and cladocerans (asexually vs. sexually reproducing populations) we hope to establish the relative roles of genetic and epigenetic mechanisms in determining the adaptation capacity of marine populations to pCO_2_. The results from this study provide a basis for investigating the possibility for rapid adaptive responses of planktonic marine organisms to climate change. Until recently, studies addressing the epigenetic basis of adaptation have been conducted mainly in sessile animals such as corals and mollusks.

## 2 MATERIALS AND METHODS

### 2.1 Animal collection and experimental design

Animals (*A*. *clausi* and *E*. *nordmanni*) were collected at the Austevoll Research Station, Institute of Marine Research, Norway (60.086 N, 5.262 E) on 6–10 October 2018 using a 0.75 m diameter, 333 μm mesh zooplankton net towed at speeds of <1 kn. Upon returning to the lab, samples were sorted immediately and maintained in 330 μm filtered seawater overnight (~12 h) before being transferred to the experimental conditions. The experiment was conducted at 2 *p*CO_2_ concentrations (500 ppm and 1,200 ppm CO_2_ Table 1) based on IPCC (2021) projected end of century pCO_2_ concentrations under model scenarios SSP5‐8.5 and SSP2‐4.5.

**TABLE 1:** Mean carbonate chemistry measured prior to the 24 h incubation

Species	pCO_2_	AT	pH	NO_2_	NO_3_	PO_4_	Si	CT	HCO_3_	${\mathrm{CO}}_{3}^{2‐}$	Calculated pCO_2_	Ω Ar	Ω Ca
		μmol/kg	Total	μmol/kg	μmol/kg	μmol/kg	μmol/kg	μmol/kg	μmol/kg	μmol/kg	μatm		
Acartia	Low	2,293 ± 3	7.989 ± 0.011	0.16 ± 0.04	11.3 ± 2.6	1.09 ± 0.44	6.0 ± 0.2	17.9 ± 0.5	1,960 ± 7	134.1 ± 2.8	455 ± 14	2.06 ± 0.04	3.21 ± 0.07
	High		7.688 ± 0.007					38.5 ± 0.6	2,113 ± 3	72.5 ± 1.0	983 ± 16	1.11 ± 0.01	1.74 ± 0.02
Evadne	Low	1,850 ± 3	7.911 ± 0.009	0.44 ± 0.04	3.2 ± 0.3	0.30 ± 0.08	6.5 ± 2.5	19.6 ± 0.5	1,652 ± 4	78.6 ± 1.5	471 ± 11	1.23 ± 0.02	1.95 ± 0.04
	High		7.623 ± 0.005					40.3 ± 0.5	1,743 ± 1	42.5 ± 0.5	964 ± 12	0.66 ± 0.01	1.06 ± 0.01

The system used to augment the CO_2_ levels of the seawater is described in Runge et al (2019). Briefly, seawater is pumped from the Bjørnafjord at a depth of 160 m up to the laboratory facilities, where it is sand‐filtered then passed through a 20 mm Arcal disk filter. This seawater input is then split between a set of 100 L mixing tanks and a stock solution tank, that is, bubbled with CO_2_ to maintain a pH of 5.8. Dosing pumps (Iwaki Inc.), controlled by feedback from pH electrodes and controllers (Endress and Hauser, Liquiline CM 442), then add the low‐pH stock solution to the mixing tanks to create seawater at treatment pCO_2_ levels (Table 1).

Animals were placed in 250 ml culture flasks filled with water from this system and held in a temperature‐controlled environmental chamber at 12.5°C (±0.1°C) for 24 h. Experiments were run with four replicate chambers with ~20 *E*. *nordmanni* or ~40 *A*. *clausi* in each chamber. Full carbonate chemistry of the water was measured prior to exposure and pH was measured after exposure. Given the sealed environments and limited exposure time, electrode measurements at the end of 24 h showed that pH never increased by more than 0.068 units in any replicate, averaging a 0.027 unit increase from the initial values. At the end of the experiment, animals were filtered from each chamber and pipetted into 3 ml cryotubes and frozen at −80°C until analyzed for gene expression and DNA methylation profiles.

### 2.2 Carbonate chemistry

Carbonate chemistry was calculated from the total alkalinity (AT; measured by titration) and from pH. The pH (total) was measured spectrophotometrically (Hitachi U‐2900 dual‐beam) using the pH‐sensitive indicator dye m‐cresol purple (Sigma‐Aldrich) following SOP (standard operating procedure 6b: (Dickson et al., 2007)). Samples of seawater were collected in 20 ml scintillation vials (leaving no head space) from all experimental vessels and held in a dark, 25°C water bath for temperature equilibration. pH was always measured within 3–5 h of sample collection. To make each pH measurement, 10 ml of each sample was slowly pipetted into two quartz cuvettes with a 5 cm path length (a modification of the 10 cm path length in SOP 6b). The cuvettes were sealed with a Teflon cover, and held at 25°C in the temperature‐controlled chamber of the spectrophotometer. M‐cresol purple (10 μl) was added to the sample cuvette, while the second cuvette served as a reference. Absorbance was measured at 578 nm (A1), 434 nm (A2), and 730 nm (background). We used equations in section 8.3 of SOP 6b to correct A1/A2 for the addition of dye. The pK2 and final pH value was determined from Liu et al. (2011, Equation 18). Carbonate chemistry was determined from pH, total alkalinity (AT), temperature, salinity, and nutrients (phosphate and silicate). AT was analyzed by potentiometric titration (Dickson et al., 2007) in an open cell with 0.1 M HCl using a Hach AT1122[SS1] automatic titrator (Loveland, CO, USA). Certified reference material provided by Andrew Dickson (Scripps Institution of Oceanography, San Diego, USA) was used to calibrate AT measurements. An additional sample (20 ml) was collected and stored in HDPE bottles with HDPE caps with 200 μl of chloroform added. These samples were then analyzed for silica, phosphorus, and nitrogen. Additional carbonate chemistry parameters were calculated using CO2SYS2.3 (Lewis et al., 1998) with the standard set of carbonate system equations and constants of (Mehrbach et al., 1973) after applying the refit of (Dickson & Millero, 1987).

### 2.3 Transcriptome profiling

**FIGURE 1:**
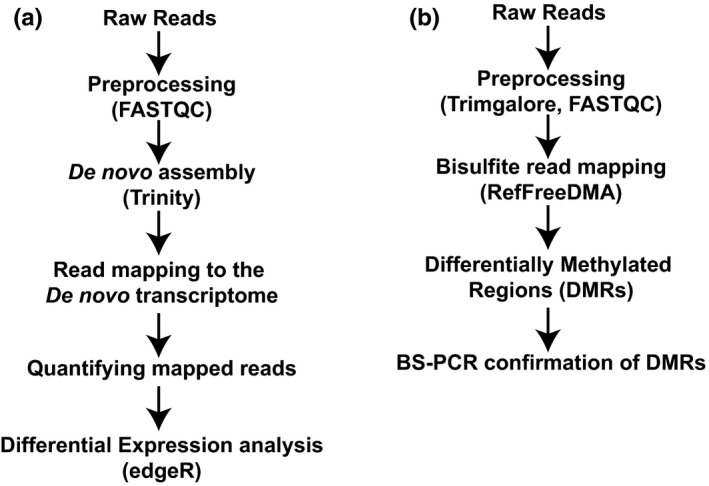
Analysis pipeline used for transcriptomic (a) and DNA methylome (CpG; b) analysis. Transcriptome and DNA methylation analysis was conducted following established pipelines (details described in the methods section)

Unstranded RNAseq libraries for both the species were prepared using the Illumina TruSeq total RNA library prep kit and 50 bp single‐ends sequencing on the HiSeq2000 platform were performed at the Tufts University Core Facility. Raw data files were preprocessed by trimming the reads using Trimmomatic (Bolger et al., 2014), removing the low quality reads (Phred score <35) and assessed for quality using FastQC (Andrews, 2010). Preprocessed reads were concatenated and *de novo* transcriptome assembled using Trinity (v.2.8.6, (Haas et al., 2013)) (Figure 1a). We used default parameters except for minimum contig length (set to 300) and normalize maximum read coverage (set to 50). Functional annotation was performed by first predicting the coding regions of the transcripts using TransDecoder (v3.0.0) (Haas et al., 2013). The predicted transcripts were annotated using BLASTx (Altschul et al., 1997). The resulting statistically significant BLAST annotations were used for Gene Ontology (GO) classification, a system for hierarchically classifying genes based on their biological process and molecular function (Ashburner et al., 2000). Differential gene expression was carried out by mapping the reads to the *de novo* assembly using kallisto (Bray et al., 2016) and statistical analysis was conducted using edgeR, a Bioconductor package (Robinson et al., 2010). GO analysis of differentially expressed genes (DEGs) was done using gProfiler. Bonferroni correction for multiple testing (*p*‐value < .05) was used while determining the fold enrichment. To understand the relationship between GO terms, Directed Acyclic Graphs of significantly enriched GO terms were drawn using GOView (webgestalt.org/GOView). Raw data files have been deposited in NCBI BioProject (PRJNA780490).

### 2.4 Quantification of global DNA methylation levels

Global cytosine levels were determined using established high performance liquid chromatography (HPLC) protocols (Ramsahoye, 2002) with some modifications. Briefly, genomic DNA was hydrolyzed with a combination of RNase A and RNase T1 followed by ethanol precipitation. DNA was digested to nucleosides as described previously (Hashimoto et al., 2015) and separated on a 6490 Triple Quad LC‐MS with UV absorbance detector (1290 Infinity UV detector, 6490 Triple Quad Mass detector, Agilent, Santa Clara, CA) equipped with an XSelect™ HSS T3 column (2.1 × 100 mm, 2.5 μm, Waters, Milford, MA).

### 2.5 Enhanced RRBS and data analysis

Enhanced reduced representation bisulfite sequencing (eRRBS) library preparation and sequencing was conducted by ZymoResearch. Briefly, libraries were prepared from 200 to 500 ng of genomic DNA digested with 60 units of TaqαI and 30 units of MspI (New England Biolabs, MA) sequentially and then extracted with DNA Clean and Concentrator™‐5 kit (ZymoResearch, CA). Fragments were ligated to preannealed adapters containing 5′‐methylcytosine instead of cytosine according to Illumina's specified guidelines. Adaptor‐ligated fragments of 150–250 bp and 250–350 bp in size were recovered from a 2.5% NuSieve 1:1 agarose gel (Zymoclean™ Gel DNA Recovery Kit). The fragments were then bisulfite‐treated using the EZ DNA Methylation‐Lightning™ Kit. Preparative‐scale PCR was performed and the resulting products were purified and 50 bp paired end (PE) sequencing was performed on an Illumina HiSeq2500 platform. Sequence reads from eRRBS libraries were identified using standard Illumina base‐calling software. Raw reads were preprocessed using TrimGalore and aligned to the genome using RefFreeDMA (Klughammer et al., 2015). RefFreeDMA was designed for conducting differential DNA methylation analysis with deduced reference genome, thus allowing DNA methylation profiling in organisms without a reference genome (Figure 1b). We conducted DNA methylation analysis only in the CpG context. We did not conduct the bisulfiteBlast step in RefFreeDMA pipeline to verify species annotations due to several issues in modifying the provided script for our experimental dataset. Raw data files have been deposited in NCBI BioProject (PRJNA780490).

### 2.6 Bisulfite PCR (BS‐PCR)

Methylation analysis of CpG islands was performed by BS‐PCR. A 50 μl PCR reaction was carried out in 1X PCR buffer, 5 mM MgCl_2_, 1 mM dNTP mix, 1 unit of Taq polymerase, 50 pmol each of the forward and reverse primers, and ~50 ng of bisulfite‐treated genomic DNA. BS‐PCR primers were designed using the sense strand of the bisulfite‐converted DNA. PCR cycling conditions were 94°C for 10 min, followed by 40 cycles of (94°C for 30 s, 55°C for 30 s and 72°C for 30 s), and a final cycle of 72°C for 8 min. PCR products were electrophoresed on 1% agarose gels, bands excised and gel‐extracted using the Gene Clean II kit (MP Biomedical, CA). Purified PCR products were cloned using the pGEM‐Teasy cloning kit (Promega, MI) as per the manufacturer's protocol. Mini‐preps were prepared using the Pure Yield plasmid miniprep kit (Promega, MI). The primer sequences are provided in Table S1. For each sample, a minimum of 5 clones were sequenced. BS‐PCR together with sequencing of several clones provides allele‐specific methylation profiles.

## 3 RESULTS

Calculated pCO_2_ values in the low pCO_2_ exposures ranged from 455 (14 SD) to 552 (31 SD) μatm (Table 1). Spectrophotometrically measured pH values in the low pCO_2_ treatments ranged from 7.856 (0.022 SD) to 7.989 (0.011) (Table 1). These values are well within the range of current surface seawater conditions (IPCC, 2013). In the high pCO_2_ treatments values ranged from 983 (16 SD) to 1089 (20 SD) μatm with a corresponding pH value that ranged from 7.602 (0.003 SD) to 7.688 (0.007 SD). These values correspond to projected pCO_2_ and pH values for the year 2150 assuming “business as usual” scenarios (IPCC, 2013) and model scenarios SSP5‐8.5 for end of the century (IPCC, 2021).

### 3.1 Transcriptome profiling

Illumina sequencing of *A*. *clausi* and *E*. *nordmanni* libraries yielded an average 250 million single end reads, 85% of which were high quality reads. The assembly produced 55,322 and 43,234 transcripts with open reading frames in *A*. *clausi* and *E*. *nordmanni*, respectively (Table 2). Approximately 80% of these transcripts are singletons and a majority of them (~60%–65%) are annotated with either GO biological process or molecular function terms.

**TABLE 2:** Basic *de novo* assembly and annotation statistics of *A*. *clausi* and *E*. *nordmanni* transcriptomes

	*A. clausi*	*E. nordmanni*
Raw reads	276,171,339	235,819,945
Processed reads	236,232,424	199,323,424
Assembly statistics		
Transcripts	189,233	142,242
Trinity predicted genes	143,423	98,343
Unique TR identifiers	98,676	67,788
Minimum sequencing length (bp)	342	321
Average contig length	987	665
GC content (%)	44	41
N50 (bp)	1,293	987
Number of mapped reads	197,342,211	181,435,219
Transcript annotation		
Transcripts with coding regions	55,322	43,234
Transcripts with BLAST hits	46,434	35,868
GO terms	47,343	35,678

**FIGURE 2:**
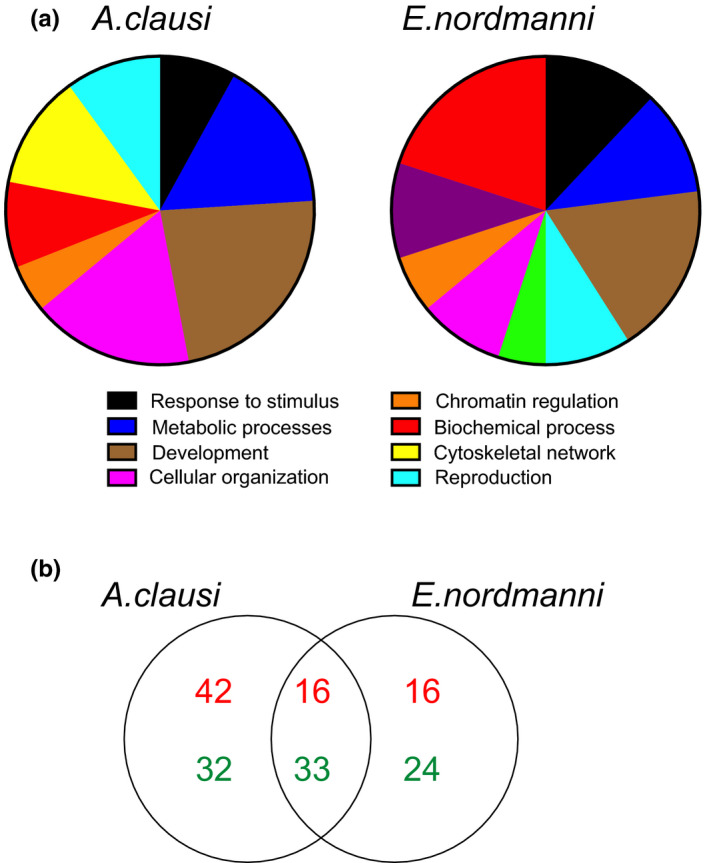
*A*. *clausi* and *E*. *nordmanni* transcriptomes and their response to OA exposure. (a) Representation of major GO terms represented in *de novo* transcriptomes of *A*. *clausi* and *E*. *nordmanni*, respectively. (b) The number of DEGs in response to OA (900 ppm) exposure in *A*. *clausi* and *E*. *nordmanni*. Both unique and common sets of DEGs are shown in the venn diagram. False discovery rate of 5% was used as a statistical cutoff. The complete list of DEGs is provided in Table S1

Differential gene expression analysis revealed OA‐induced changes in gene expression in both of the species investigated. In *A*. *clausi*, exposure‐dependent changes in gene expression were observed. In response to high pCO_2_ exposure 123 genes were differentially expressed (Figure 2). GO analysis of DEGs demonstrated enrichment of GO terms such as energy reserve metabolic process (GO:0006112), response to hypoxia (GO:0001666), cytoskeleton (GO:0005856) and extracellular matrix organization (GO:0030198) (adjusted *p* value <.05). In addition, GO term—one carbon metabolic process (GO:0006730) was represented, although it was not significant (adjusted *p* value = .067).

In *E*. *nordmanni*, we observed 89 DEGs in response to high pCO_2_ exposure (Figure 2). GO term analysis revealed enrichment of terms such as mitochondrion organization (GO:0007005), NADH dehydrogenase complex (GO:0010257), mitochondrial respiratory chain complex (GO:0032981), one carbon metabolic process (GO:0006730), hypoxia and transcription factor activity (adjusted *p* value < .05).

**FIGURE 3:**
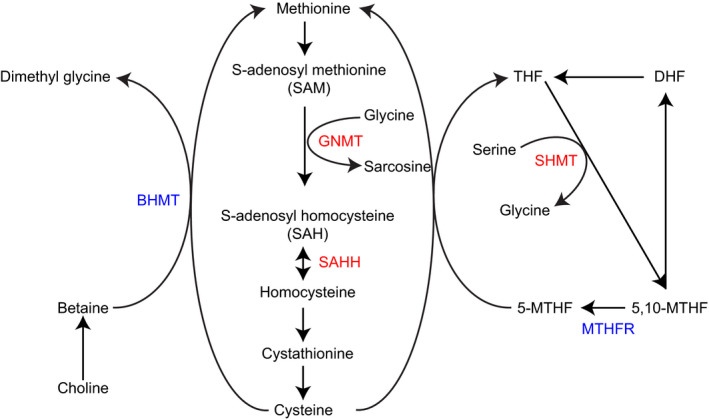
Schematic representation of one‐carbon metabolism pathway. Five genes encoding important enzymes in this pathway are differentially expressed in response to OA exposure. The genes highlighted in red and blue are up‐ and downregulated, respectively. Both of these genes are differentially expressed in *A*. *clausi* and *E*. *nordmanni*. BHMT, betaine‐homocysteine S‐methyltransferase; GNMT, glycine N‐methyltransferase; SAHH, S‐adenosylhomocysteine hydrolase; SHMT, serine hydroxymethyltransferase; MTHFR, methylenetetrahydrofolate reductase

We compared the DEGs observed in response to high pCO_2_ exposure in both species and observed 49 DEGs to be commonly expressed. Among them, 16 genes were upregulated and 33 downregulated. GO analysis suggests that many of these genes fall under the GO term one carbon metabolic process (GO:0006730), oxidative phosphorylation (GO:0006119), and NADPH regeneration (GO:0006740). We observed five DEGs related to the GO term—one carbon metabolic process in both species (Figure 3).

### 3.2 Global and genomewide DNA methylation levels

**FIGURE 4:**
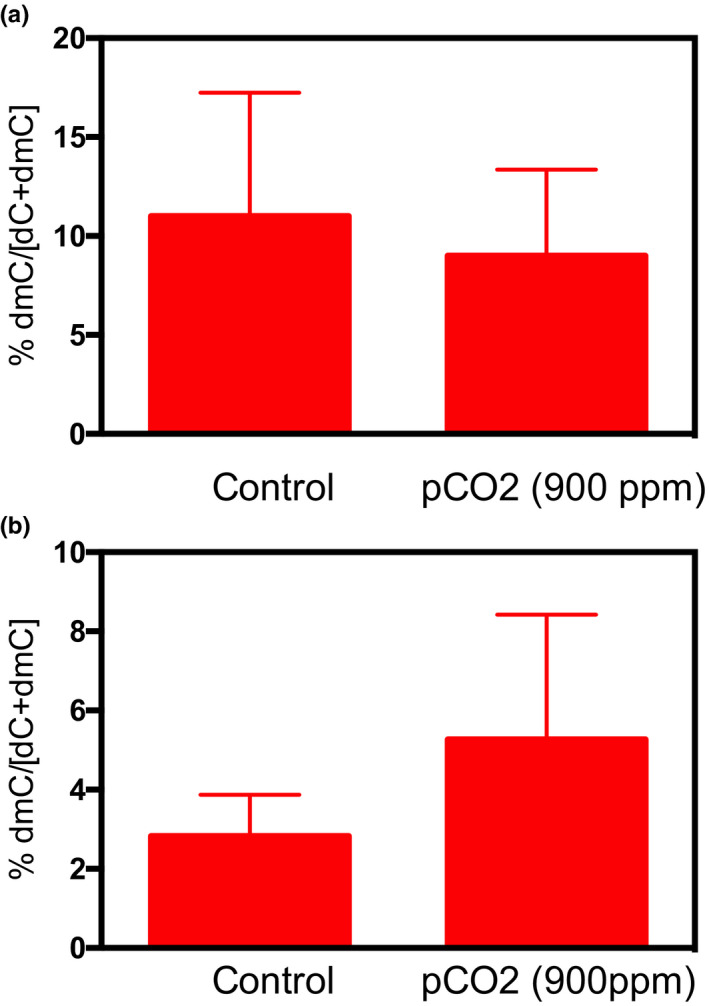
Global 5‐methylcytosine levels in response to OA measured by the HPLC method in (a) *A*. *clausi* and (b) *E*. *nordmanni*. All values represent mean + standard error of mean (SEM; *n* = 4). Fisher's *t*‐test was used to determine the effect of treatment. No statistically significant effects were observed

**FIGURE 5:**
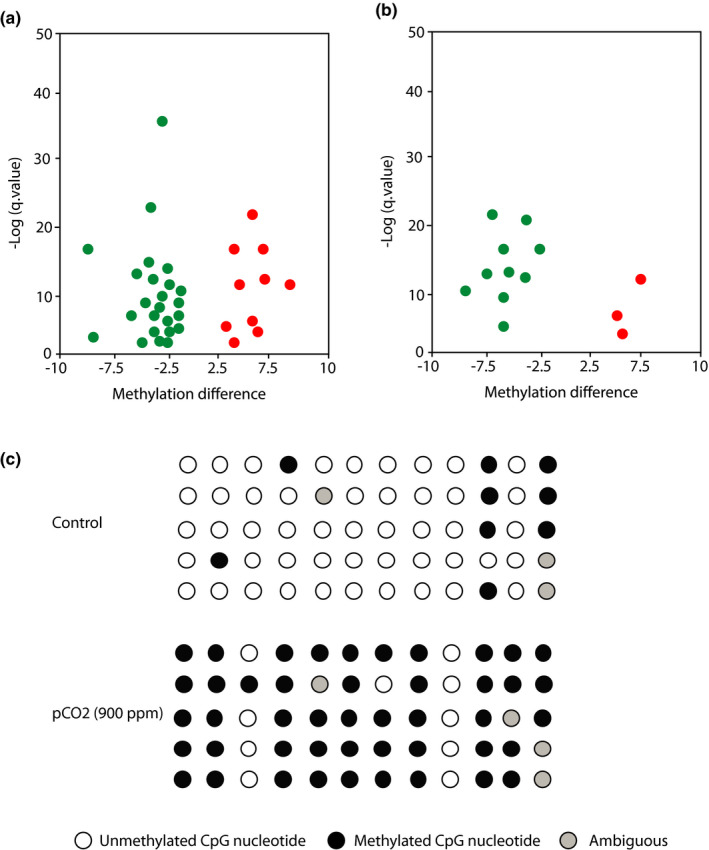
DNA methylation profiling in *A*. *clausi* and *E*. *nordmanni* (a and b). Volcano plots showing hypo and hypermethylated differentially methylation regions in response to OA exposure. Percent methylation difference (x‐axis) between *p*CO_2_ (900 ppm) and Control are plotted against q‐value (y‐axis). *Q*‐value of 0.05 was used as a statistical cutoff in differential methylation analysis. Each green and red spot represents a statistically significant hypo and hypermethylated region, respectively. (c) Confirmation of a differentially methylated region observed in response to *p*CO_2_ (900 ppm) in *A*. *clausi*. Lollipop diagram showing the differences in methylation status between *p*CO_2_ (900 ppm) and control. Each row represents a sequenced clone. Each lollipop represents one CpG dinucleotide. Filled and open circles denote methylated and unmethylated sites, respectively. Ambiguous sites are represented in gray color

OA exposure did not affect 5‐methylcytosine levels measured by HPLC (Figure 4). Using RRBS, we sequenced an average of 34 million and 31.66 million paired‐end reads from *A*. *clausi* and *E*. *nordmanni* samples, respectively. The bisulfite conversion efficiency was 90%–93%. The mapping efficiency of these reads to the bisulfite converted assembled genome was between 36% and 38%, which is comparable to previously published studies in other species (Aluru et al., 2018; Chatterjee et al., 2012; Jeremias et al., 2018). On average, 112 million cytosines were sequenced. Of these 8.5 and 15.2 million were methylated and unmethylated, respectively, in a CpG context. In both species, we sequenced approximately one million unique CpGs per sample with 1× coverage. Principal component analysis shows clustering of samples based on treatment conditions (Figure 5a). We observed 34 and 14 differentially methylated regions (DMRs) in *A*. *clausi* and *E*. *nordmanni*, respectively. Among the 34 DMRs in *A*. *clausi*, 25 of them are hypomethylated and 9 DMRs are hypermethylated. Similarly, 11 out of 14 DMRs in *E*. *nordmanni* were hypomethylated and the remaining 3 were hypermethylated in response to OA exposure (Figure 5b).

## 4 DISCUSSION

OA induced changes in the transcriptome and DNA methylome of both of the species studied. Transcriptome profiling revealed a high degree of conservation in OA‐induced changes in gene expression among these marine invertebrates. In addition, DNA methylation profiling revealed subtle but specific changes in CpG methylation in response to OA exposure.

At the organismal level, *A*. *clausi and E*. *nordmanni*, are tolerant of *p*CO_2_ levels and temperature increases relevant for future OA scenarios (Zervoudaki et al., 2013). In this study, significant transcriptional responses were observed by transcriptional profiling. Several studies have investigated the effect of OA on the transcriptome in a number zooplankton species (Beszteri et al., 2018; Johnson & Hofmann, 2017; Strader et al., 2020). Even though the experimental conditions are highly variable between these studies, there are some consistent transcriptional responses observed in multiple species. One of the well documented changes includes differential expression of genes associated with metabolism. We observed similar responses in both species. This is not surprising given the fact that elevated carbon dioxide causes metabolic depression (Michaelidis et al., 2005; Pörtner et al., 1998; Reipschläger & Pörtner, 1996). This mainly results from a decrease in extracellular pH and compensatory mechanisms exist to re‐establish the acid‐base regulation. We observed differential expression of genes encoding ion transporters, suggesting that mechanisms associated with metabolic depression are highly conserved.

Another widely demonstrated effect of high CO_2_ exposure is altered fatty acid and protein metabolism (Diaz‐Gil et al., 2015; Leu et al., 2013; Mayor et al., 2015). Similar to previous studies, high CO_2_ exposure caused downregulation of genes associated with lipid and protein metabolism in both species. The physiological effects of altered expression of these genes in response to acute high CO_2_ exposure have not been investigated. Our results suggest that under acute stress organisms undergo budget reallocation of energy reserves and reduce energetically expensive metabolic processes. This has been demonstrated in several species where acute exposure to stressors leads to metabolic reorganization and saving of energetic reserves for maintenance of basal metabolism (McLaskey et al., 2019; Van de Waal & Litchman, 2020).

The consequences of metabolic depression and other metabolic changes observed range from developmental delay to effects on metamorphosis and growth. While these changes may be compensatory and/or adaptive responses to acute stress, chronic effects could have detrimental effects on reproduction, and fitness. Several studies have investigated multigenerational effects of OA and the results suggest that acute exposure to high CO_2_ could result in the development of tolerance to subsequent exposures, in some cases through multiple generations (Donelson et al., 2012; Sunday et al., 2014). However, these results are species‐specific. It is widely established that persistent changes in physiology or gene expression, that is, induced by a previous exposure to stressors are considered an "epigenetic" memory (Jeremias et al., 2018). This memory is based on DNA methylation and histone modifications that alter chromatin accessibility and influence gene transcription. Very few studies have investigated the role of epigenetic memory in environmental adaptation to stressors in nonmodel invertebrate species. One of the GO pathways that was enriched in the RNAseq dataset is the one‐carbon metabolism pathway. The substrates from one‐carbon metabolism play an important role in the maintenance of cellular nutritional status by converting nutrients (e.g., glucose, amino acids) into metabolites that feed into diverse biological functions, including cellular biosynthesis, maintaining cellular redox status. In addition, it provides substrates involved in the regulation of protein and nucleic acid methylation (Locasale, 2013). The observed CO_2_ exposure related differences in one‐carbon metabolic pathway suggest that cellular metabolism is impacted. This could potentially affect epigenetic regulation of gene expression, nucleic acid biosynthesis and metabolic disturbances, something that should be investigated in future studies.

Global 5‐methyl cytosine profiling of DNA using two different methods did not reveal any significant changes in response to high pCO_2_ exposure in either of the species. However, genome wide profiling identified several differentially methylated regions with two‐thirds of them hypomethylated in response to OA. While these results suggests that methylation or demethylation may have an important role in the organisms response to OA, the lack of a well annotated genomic resource for these species makes assigning differentially methylated regions to specific genes impossible. Future work in this arena is certainly warranted.

In recent years, the belief that the genetic code is the sole basis for biological inheritance has been challenged by the discovery of trans‐generational epigenetic inheritance. Through epigenetics, environmentally induced phenotypes can persist for several generations, due to the transmission of molecular factors that determine how DNA is read and expressed (Bonduriansky & Day, 2009; Jablonka & Raz, 2009; Verhoeven et al., 2016). While epigenetic inheritance is well documented (Verhoeven et al., 2016), the adaptive significance, if any, of such a complementary inheritance system remains enigmatic. Since it constitutes the inheritance of an environmentally induced phenotype, its adaptive value should depend upon whether environments are predictable across generations. The use of clonal species such as cladocerans provides a promising avenue for determining the degree of heritability and the longevity of epigenetic changes in subsequent generations.

## CONFLICT OF INTEREST

The authors have no conflict of interest to declare.

## AUTHOR CONTRIBUTIONS

**Neelakanteswar Aluru:** Conceptualization (equal); data curation (lead); formal analysis (lead); funding acquisition (supporting); investigation (equal); methodology (lead); project administration (supporting); resources (supporting); software (lead); supervision (supporting); validation (lead); visualization (lead); writing–original draft (lead); writing–review and editing (lead). **David M. Fields:** Conceptualization (lead); data curation (supporting); formal analysis (supporting); funding acquisition (lead); investigation (lead); methodology (lead); project administration (supporting); resources (equal); software (supporting); supervision (lead); validation (supporting); visualization (supporting); writing–original draft (equal); writing–review and editing (equal). **Steven Shema:** Conceptualization (supporting); data curation (supporting); formal analysis (supporting); funding acquisition (supporting); investigation (equal); methodology (equal); project administration (supporting); resources (supporting); software (supporting); supervision (supporting); validation (supporting); visualization (supporting); writing–original draft (supporting); writing–review and editing (supporting). **Anne Berit Skiftesvik:** Conceptualization (equal); data curation (supporting); formal analysis (supporting); funding acquisition (equal); investigation (equal); methodology (equal); project administration (equal); resources (equal); software (supporting); supervision (equal); validation (supporting); visualization (supporting); writing–original draft (supporting); writing–review and editing (supporting). **Howard I. Browman:** Conceptualization (lead); data curation (equal); formal analysis (supporting); funding acquisition (lead); investigation (lead); methodology (equal); project administration (lead); resources (lead); software (equal); supervision (lead); validation (equal); visualization (equal); writing–original draft (equal); writing–review and editing (equal).

## Supporting information

Table S1

## Data Availability

High throughput sequencing raw data (RNA sequencing and DNA methylation) has been deposited in NCBI BioProject (Accession number: PRJNA780490).
