# Supplementary material for: Gene expression and epigenetic responses of the marine Cladoceran, Evadne nordmanni, and the copepod, Acartia clausi, to elevated CO2
Source: Ecol Evol. 2021 Nov 23;11(23):16776–85. doi: 10.1002/ece3.8309 (PMC8668794; doi:10.1002/ece3.8309)
Supplement: Supplementary file 1 — Table S1 [file ECE3-11-16776-s001.docx]

Table S1. Primer sequences used for amplifying a randomly selected differentially methylated region (DMR_15) from *Acartia clausii* for confirmation using bisulfite PCR (BS-PCR).

| Primer | Sequence (5'-3') | Annealing Temperature (^o^C) |
| --- | --- | --- |
| Forward | CAGTCCGCGGATCATTGGCCGATCCC | 64 |
| Reverse | GGGCCTAATTCCCGGGTTAACCTGCGGG |  |
